# The complete genome sequence of the cluster BE1 *Streptomyces Bacteriophage Riptide* includes genes encoding ribosome-associated proteins

**DOI:** 10.1128/mra.00776-25

**Published:** 2025-09-12

**Authors:** Alena Sagalovsky, Aniket Camarushi, Jeshuwin D. Prabakaran, Abdullah Shahzad, Nicholas Nguyen, Daniel Mirahmadian, Michelle A. Ibino, Ali Mutasim, Emmanuel Okusanya, Choudhury Zaki, Kimia Kardaniyazd, Hope Ramsey, Jariatu Kargbo, Beryl Fuamazeh, Dania Mahmood, James Anderson, Eni Adesola, Laure Elisabeth Fom Kembou, Cameron Hindle, Keyshawn Kontchou, Abdullah Najib, Gilbert Hernandez, 2024 UMBC Phage Hunters, Marie-charlotte Adoni-Sedjro Agbogbe, Elana Frazier, Steven M. Caruso

**Affiliations:** 1 Department of Biological Sciences, University of Maryland, Baltimore County 14701, Baltimore, Maryland, USA; Portland State University, Portland, Oregon, USA

**Keywords:** bacteriophage, *Streptomyces*, phage, genomics, metal tolerance

## Abstract

This study isolated bacteriophage *Riptide*, a BE1 cluster siphovirus, from soil using *Streptomyces mirabilis* NRRL B-2400. *Riptide* follows a lytic life cycle contains a genome length of 132,142 bp encoding 236 protein coding genes including two with ribosomal-related functions, as well as genes that encode 39 tRNAs and 1 tmRNA.

## ANNOUNCEMENT

The gram-positive spore-forming bacterial genus *Streptomyces* is known for producing antibiotics and other secondary bioactive metabolites (1). Studying *Streptomyces*-infecting bacteriophages can provide a clearer picture of the genera’s diversity, evolution, and bacteriophage resistance (1).

**Fig 1:**
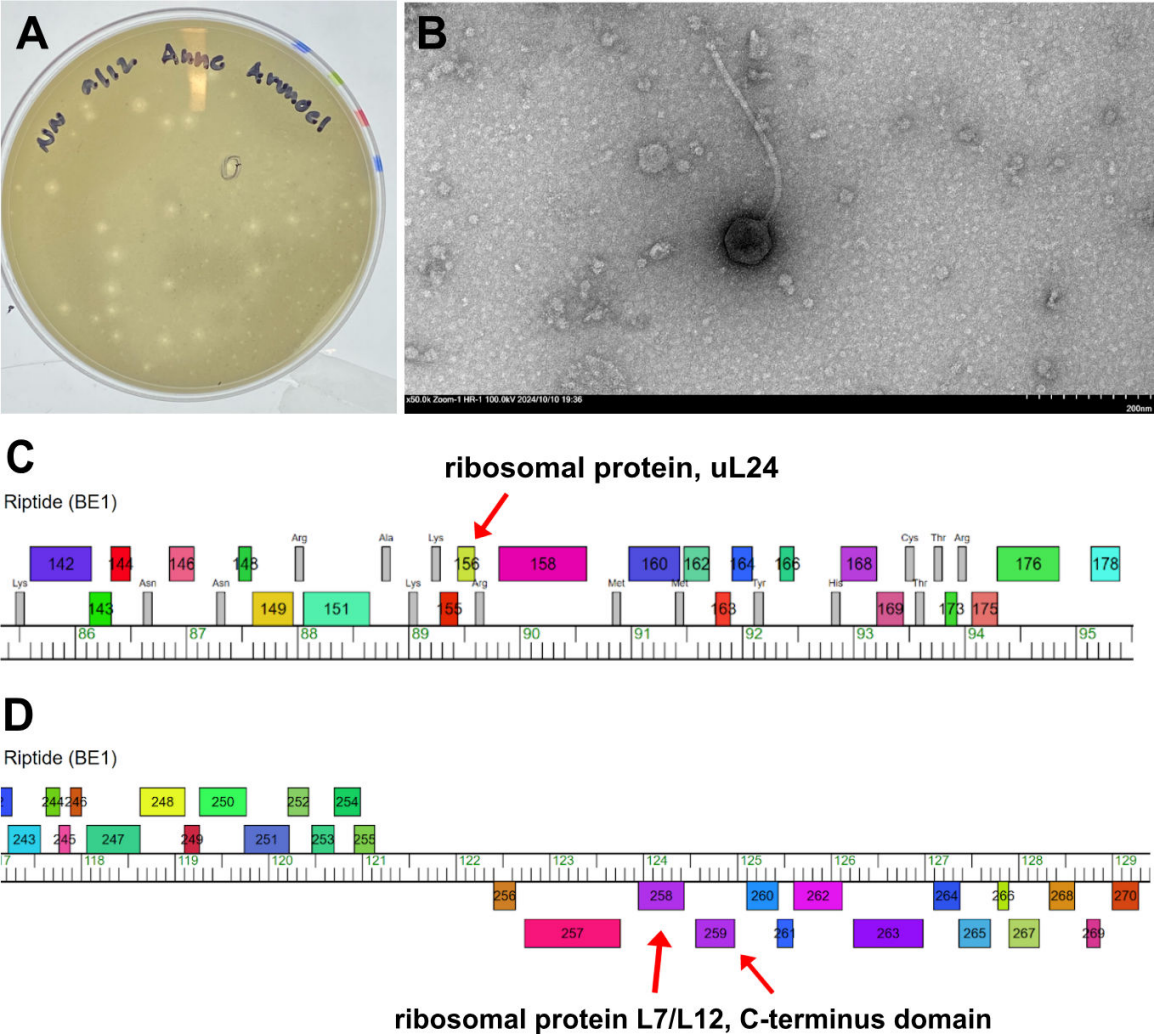
(**A**) *Riptide* plaque morphology. Plaques exhibit consistent round and turbid morphology, with an average 3 mm diameter. (**B**) *Riptide* particle morphology. A fresh lysate was analyzed via transmission electron microscopy (TEM), identifying *Riptide* as having a siphoviral morphotype. ImageJ was used to scale the image and obtain the head and tail measurements using measurements from three particles (3). The phage capsid diameter was 75 nm, tail length was 328 nm, and tail width was 11 nm. (C/D) Genome map of *Riptide* genome created in Phamerator of regions containing ribosome-associated genes. Boxes represent identified genes with genes producing proteins in the same protein family (pham) assigned the same color (4). Selected predicted functional domains are annotated above the genome. (**C**) Gene 156 (indicated with arrow) has been assigned the function “ribosomal protein, uL24.” (**D**) Genes 258 and 259 (indicated with arrows) have been assigned functions “ribosomal protein L7/L12, C-terminus domain.”

*Streptomyces phage Riptide* was isolated from 3 g of soil collected on 9/4/24 from a garden in Odenton, Maryland (GPS: 39.069053 N, 76.724572 W) using the host *Streptomyces mirabilis* NRRL B-2400 (*S. mirabilis*) using protocols described previously (2). The soil was washed in a buffer of 10 mM Tris, 10 mM MgSO_4_, 68 mM NaCl, and 1 mM CaCl_2_, and the resulting solution was filtered (0.22 µm). The sample was combined with a 48 h culture of *S. mirabilis* grown in nutrient broth (BD Difco) supplemented with MgCl_2_, Ca(NO_3_)_2_, and glucose, plated on nutrient agar using tryptic soy top agar (BD), and incubated at 30°C for 24–48 h. Three rounds of plaque purification were used to isolate the phage. Plaques averaged ~3 mm in diamete were and turbid when grown on the isolating host as tested (Fig. 1A). A freshly harvested lysate from a plate containing confluent plaques (2) was produced and negative stained using uranyl acetate for transmission electron microscopy (TEM). Analysis by TEM revealed *Riptide*’s siphoviral morphology with a 75 nm capsid diameter and 328 nm tail length (Fig. 1B).

Double-stranded DNA was isolated from lysate harvested the same day using the Promega Wizard DNA Cleanup kit. DNA was prepared using the NEB Ultra II Library Kit and sequenced using an Illumina NextSeq 1000 to yield 2,167,332 single-end 100 bp reads. Reads were trimmed and filtered by Cutadapt 4.7 (5) and Skewer 0.2.2 (6) assembled with ~1,733× coverage with Unicycler v0.5.0 (7) and checked for completeness using Consed v29 (8) as described (9). *Riptide* has a circularly permuted genome of 132,142 bp and GC content of 49.6%. *Riptide* was assigned to phage subcluster BE1 based on nucleotide and genomic similarity (10) as identified in the Actinobacteriophage database (11). Riptide belongs in the ICTV (https://ictv.global/) designated genus *Samistivirus* (12). Positional annotations were completed using DNA Master v5.23.6 (13), with Glimmer 3.0 and GeneMarkS v4.28 (14, 15). Annotations were manually checked using GeneMark.hmm 2.5 p using S. scabiei 87_22 as the model (16), Starterator v1.2 (17), and BLASTp (18). Functional annotations were completed with BLASTp, HHpred (19), and Phamerator (4). Aragorn v1.2.38 (20) and tRNAscan-SE v2.0 (21) were used to identify tRNA genes. Default parameters were used except where otherwise noted.

Annotation of *Riptide* resulted in the functional gene assignment “ribosomal protein, uL24” (Fig. 1C) and “ribosomal protein, L7/L12 C-terminus domain” (Fig. 1D) (22, 23). These functional predictions expand the known repertoire of ribosomal-related proteins in *Riptide’s* genome and suggest potential roles in translational modulation or host ribosome interaction. *Riptide* encodes a high number of tRNA genes like other cluster BE1 phages (24). *Riptide*’s GC content is significantly lower than that of its host, which typically exceeds 70%. Phage %GC correlates with host %GC (25) although several clusters of *Streptomyces* phages with very low %GC have been discussed (26).

## Data Availability

*Riptide* is available in GenBank with accession no. PV654398 and Sequence Read Archive (SRA) no. SRX28150560.
